# Combining Heat Stress with Pre-Existing Drought Exacerbated the Effects on Chlorophyll Fluorescence Rise Kinetics in Four Contrasting Plant Species

**DOI:** 10.3390/ijms221910682

**Published:** 2021-10-01

**Authors:** Lingling Zhu, Wei Wen, Michael R. Thorpe, Charles H. Hocart, Xin Song

**Affiliations:** 1 Shenzhen Key Laboratory of Marine Biological Resources and Ecological Environment, College of Life Sciences and Oceanography, Shenzhen University, Shenzhen 518060, China; eemilyling@hotmail.com (L.Z.); wenweiars@163.com (W.W.); 2 Key Laboratory of Optoelectronic Devices and Systems of Ministry of Education and Guangdong Province, College of Optoelectronic Engineering, Shenzhen University, Shenzhen 518060, China; 3 Research School of Biology, Australian National University, Canberra, ACT 2601, Australia; michael.thorpe@anu.edu.au (M.R.T.); Charles.Hocart@anu.edu.au (C.H.H.); 4 Isotopomics in Chemical Biology, School of Chemistry and Chemical Engineering, Shaanxi University of Science and Technology, Xi’an 710021, China

**Keywords:** high temperature, water deficit, photosynthesis, photobiology, fluorescence transient, electron transport

## Abstract

Although drought and high temperature are two main factors affecting crop productivity and forest vegetation dynamics in many areas worldwide, little work has been done to describe the effects of heat combined with pre-existing drought on photochemical function in diverse plant species. This study investigated the biophysical status of photosystem II (PSII) and its dynamic responses under 2-day heat stress during a 2-week drought by measuring the polyphasic chlorophyll fluorescence rise (OJIP) kinetics. This study examined four contrasting species: a C3 crop/grass (wheat), a C4 crop/grass (sorghum), a temperate tree species (*Fraxinus chinensis*) and a tropical tree species (*Radermachera sinica*). Principal component analysis showed that the combination of heat and drought deviated from the effect of heat or drought alone. For all four species, a linear mixed-effects model analysis of variance of the OJIP parameters showed that the deviation arose from decreased quantum yield and increased heat dissipation of PSII. The results confirmed, in four contrasting plant species, that heat stress, when combined with pre-existing drought, exacerbated the effects on PSII photochemistry. These findings provide direction to future research and applications of chlorophyll fluorescence rise OJIP kinetics in agriculture and forestry, for facing increasingly more severe intensity and duration of both heat and drought events under climate change.

## 1. Introduction

Drought and high temperature are two main factors affecting crop productivity and vegetation dynamics in many areas worldwide [1,2,3]. Climate models have predicted the future higher intensity and duration of both heat and droughts events [4,5], and a higher frequency for the co-occurrence of both weather extremes.

In nature, it is often the case that heat stress happens intermittently compared with drought, which persists over extended periods of time. Hence, studying heat stress in the context of pre-existing drought could provide useful information. Past studies have investigated gas exchange traits such as stomatal conductance, carbon assimilation, etc. under heat stress combined with drought [6,7,8,9,10,11]. Molecular and biochemical studies reported that drought or heat or their combination affected the expression of a range of genes and the accumulation of many species of proteins and metabolites [12,13], pointing to a complex picture that awaits further clarification/elucidation. This is required if we are to properly understand the phenomenon and thereby devise practical strategies for its amelioration. On the other hand, studies that take advantage of gas exchange measurements are valuable, as they can help yield critical information on plants’ ecophysiological responses to abiotic stresses; nevertheless, gas exchange measurements are time-consuming and the measuring conditions are stringent, limiting the applicability of this technique in many settings. Hence, applying a more convenient and flexible method that gives readily comparable data would be very helpful for rigorously monitoring and comparing plant performance across time and space.

Chlorophyll fluorescence has long been used as a convenient and sensitive indicator of stress responses in plants in various contexts, such as agriculture, forestry physiology and ecology [14,15,16]. Fluorescence rise or induction curves, usually called the OJIP test, as developed by Strasser et al. [17], have been increasingly used in various fields of plant physiology; these contain information concerning the bioenergetics and interactions between individual components of photosystem II [16,18,19,20,21,22]. This fast tool, based on non-invasive intact measurements, is advantageous in that it reveals multiple components of PSII function with a higher resolution of time-dependent dynamic responses to heat impact during drought stress. OJIP kinetics have been adopted for screening different varieties of crops subjected to heat or drought stress [18,23,24,25,26,27,28], and for tracing the senescence of rice [29] and some European oak tree species [19]. The technique links the different phases of the rise of the fluorescence signal with the redox states of multiple components of photosystem II (PSII) [16,17]. The O–J part (50 μs to 2 ms) of the fluorescence kinetic assesses the closure degree of PSII reaction centers; the J–I phase (2 to 30 ms) reflects the reduction in the secondary electron acceptor Q_B_, plastoquinone and cytochrome. The rise in the I–P part (30 ms to the peak/maximal fluorescence signal) is due to the reduction in the electron transporters of the PSI acceptor side. Abiotic stress conditions such as high temperature and drought can influence the shape of the OJIP curve in the way that they affect the light-harvesting complexes, the energy flux status of the reaction centers, and the donor and acceptor sides of PSII [16,30].

As far as the direction of the response is concerned, heat and drought stress have been reported to affect OIJP parameters in a similar manner; however, the effects of these two abiotic factors could differ in terms of degree or sensitivity. For example, heat increased the fluorescence signal at 50 μs (O phase) and reduced the peak/maximal fluorescence signal phase (P phase) [24,31,32,33,34], showing that the photosynthetic efficiency or total quantum yield had decreased. In contrast, quite a few studies reported drought alone did not decrease photosynthetic efficiency at all [9,26,35,36,37]. A K peak or K band at the early stage of the O–J phase between 200 and ~300 μs was commonly observed after heat stress [16,24,33,34]. The K peak has been proposed to indicate inhibition of the electrons donated by the oxygen-evolving complex [16,38]. Oukarroum et al. [37] also reported the presence of a K peak induced by drought. Despite these differences and similarities in the response between heat and drought, the combined effect of heat stress with pre-existing drought on OJIP kinetics remains unknown. The effects on multiple plant species that differ in growth form, such as grasses vs. trees, also remains to be determined. 

This study investigated the daily-scale chlorophyll fluorescence OJIP kinetics in response to heat stress imposed during the middle of a 2-week drought. The recovery from heat and/or drought was also examined. We studied four contrasting plant species: a C3 crop/grass (wheat), a C4 crop/grass (sorghum), a temperate tree species (*Fraxinus chinensis*) and a tropical tree species (*Radermachera sinica*). By using species differing widely in ecophysiology/morphology/phylogeny, we aimed to gain a more comprehensive understanding of how heat, when combined with pre-existing drought, affects the function of different PSII components as reflected by the OJIP kinetics in the four contrasting species. We hypothesized that the combination of heat with pre-existing drought would have greater effects on the function of different PSII components than either heat or drought alone, and that the magnitude or sensitivity would differ among species.

## 2. Results

**Figure 1:**
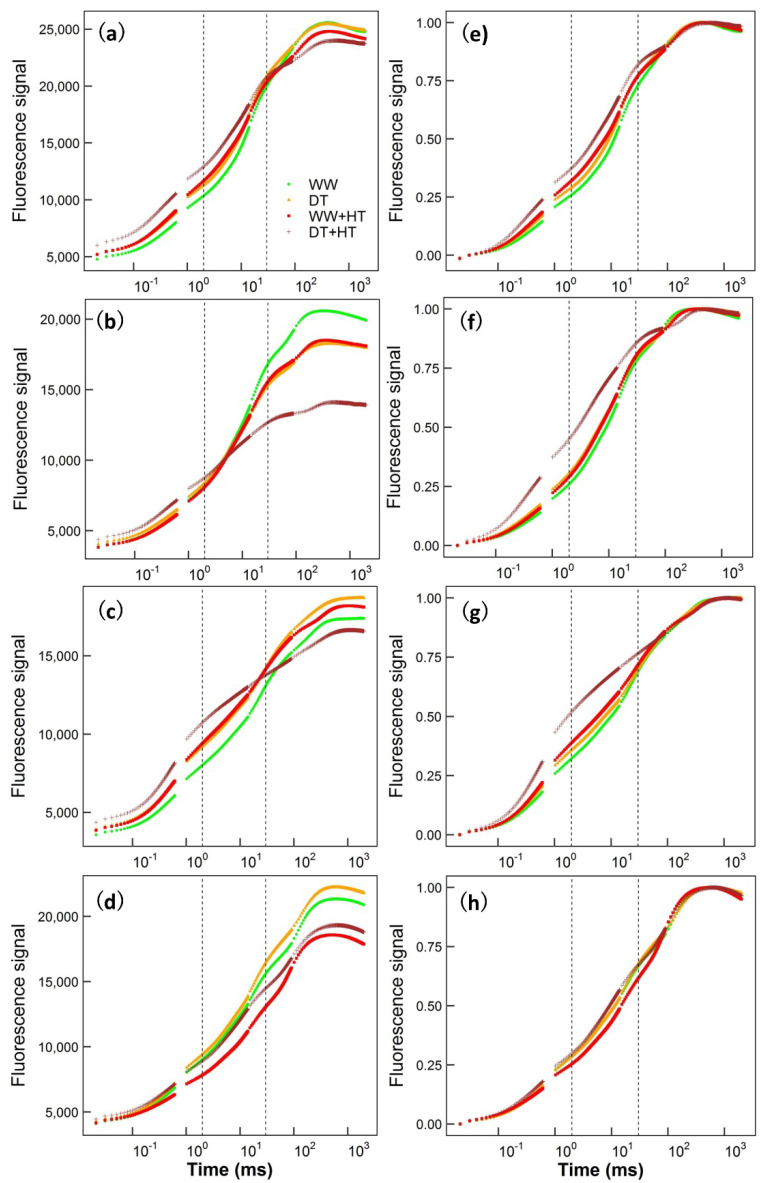
Chlorophyll fluorescence OJIP kinetic curves of four treatments in four species: wheat (**a**,**e**), sorghum (**b**,**f**), *F. chinensis* (**c**,**g**) and *R. sinica* (**d**,**h**). Panels (**a**–**d**) show the original fluorescence signal values and Panels (**e**–**h**) shows the normalized fluorescence signal values. The first vertical dashed line represents the J phase, which is at 2 ms; the second vertical dashed line represents the I phase, which is at 30 ms. “WW” and “DT” are the average of the data from the day before imposing heat stress on well-watered (WW) and droughted (DT) plants, respectively. “WW + HT” and “DT + HT” are the average of the data on the second day of heat stress in WW and DT plants, respectively.

Whole OJIP curves, acquired at a μs time resolution, of each plant species under four treatments (WW: well-watered, DT: drought-treated, WW + HT: heat stress in well-watered plants, DT + HT: heat stress in drought-treated plants) are shown in Figure 1. For the non-normalized early stage of the O–J phase (50 μs to 2 ms), which assesses the closure of some of the PSII reaction centers in response to the reduction in Q_A_, the general pattern of fluorescence magnitude was: DT + HT ≥ DT/HT ≥ WW; curves for the late stage of the I–P phase (30 ms to the peak/maximal fluorescence signal), which corresponds to the reduction in the electron transporters of the PSI acceptor side, the general pattern was: DT + HT ≤ DT/HT ≤ WW. For normalized curves, through the O–J–I phase (50 μs to 30 ms), the pattern for all species was: DT + HT ≥ DT/HT ≥ WW. Differences among treatments were smaller in the tropical tree *R. sinica* than in the other three species. The statistical analysis of the computed OJIP parameters is explained below. The curves depict a general picture that DT, HT and DT + HT changed the shape of the OJIP curves, and the magnitude differed among these treatments and among species.

**Figure 2:**
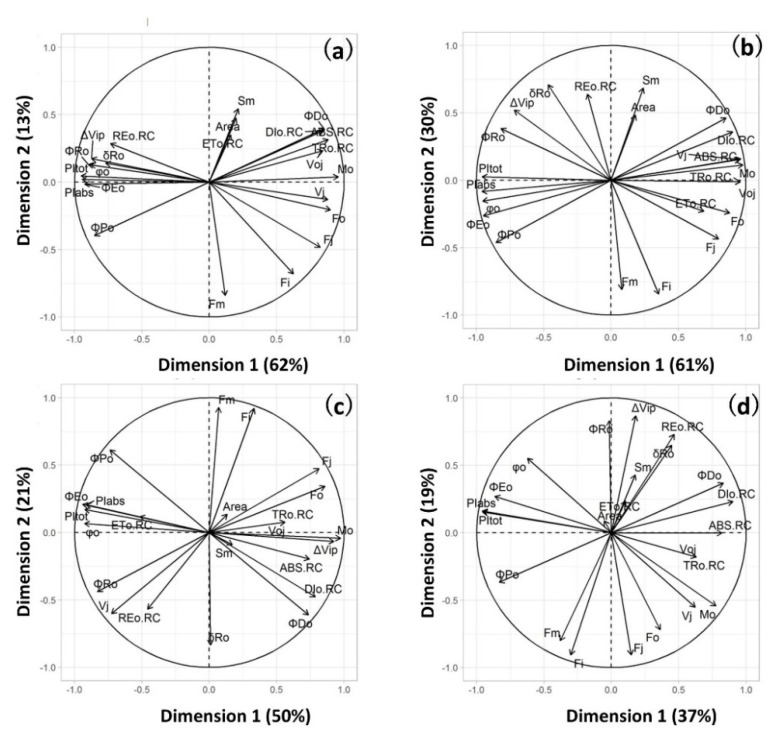
Biplots of principal component analyses of chlorophyll fluorescence OJIP kinetic parameters in wheat (**a**), sorghum (**b**), *F. chinensis* (**c**) and *R. sinica* (**d**) across all time points.

**Figure 3:**
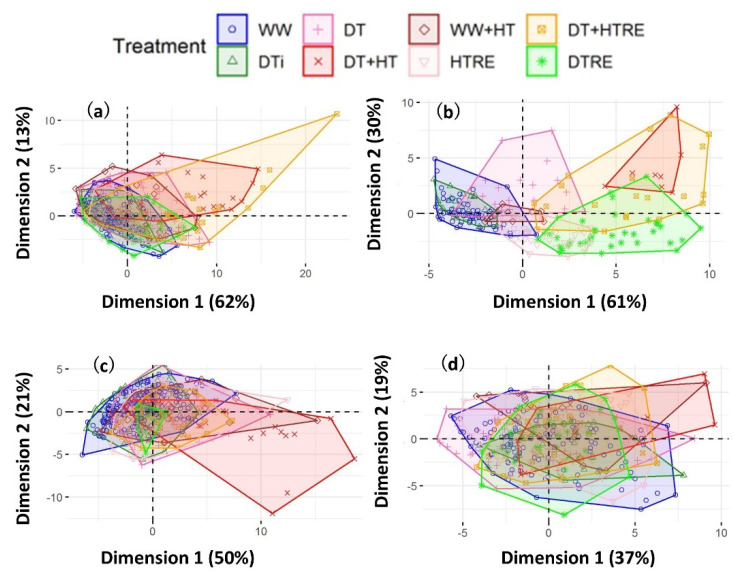
Treatment distributions on the first and second dimensions of the principal component analysis of chlorophyll fluorescence OJIP kinetic parameters in wheat (**a**), sorghum (**b**), *F. chinensis* (**c**) and *R. sinica* (**d**). WW: Well-watered; DTi: drought initiation (Day 1—the day before the start of severe drought); DT: drought (the start of targeted drought stress to the start of the heat treatment); WW + HT: well-watered plants subject to heat; DT + HT: DT plants subject to heat; DT + HTRE: heat removed from DT plants; (**d**) HTRE: WW plants removed from heat; DTRE: re-watering of DT plants.

The OJIP kinetics shown in Figure 1 were parameterized (see Table 1) and subjected to principal component analysis (PCA). For each species, PCA provides an overall picture of treatments effects, correlations between the parameters and the grouping of the treatments. we investigated. Figure 2 shows the PCA results of the OJIP parameters measured throughout the whole experimental scheme. The first dimension could explain 62%, 61%, 50% and 37% of the variation for wheat, sorghum, *F. chinensis* and *R. sinica*, respectively. The positive side of the first dimension reflects the OJIP parameters showing the increase induced by heat and/or drought stress: *F*_o_, *M*_o_, *V*_OJ_, ABS/RC, TR_o_/RC, DI_o_/RC and *ΦD*_o_. The increase in these parameters indicates the rigidity of or damage to the reaction center; less energy could be used in downstream photosynthesis, leading to more energy being dissipated as heat. The negative side of the first dimension represents the OJIP parameters showing a decrease under heat and/or drought stress (Figure 2): *ΦP*_o_, *ΦE*_o_, *ΦR*_o_, *φ*_o_, PI_abs_ and PI_tot_. The decrease in these parameters suggests the decrease in quantum efficiency or quantum yield in the light-harvesting system and the lower efficiency of energy conservation from the absorbed photons from PSII to PSI. The distribution of all treatments for each species on the first and second dimension of PCA (Figure 3) shows that, in general, DT + HT and/or DT + HTRE (recovery from heat) were oriented primarily toward the positive side of Dimension 1, followed by WW + HT and DT. In summary, the PCA shows that the combination of heat and drought led to a distinctive grouping from heat or drought alone or no stress conditions; the magnitude of the effects of heat and/or drought and recovery were greater in wheat and sorghum than in *F. chinensis* and *R. sinica*. 

**Figure 4:**
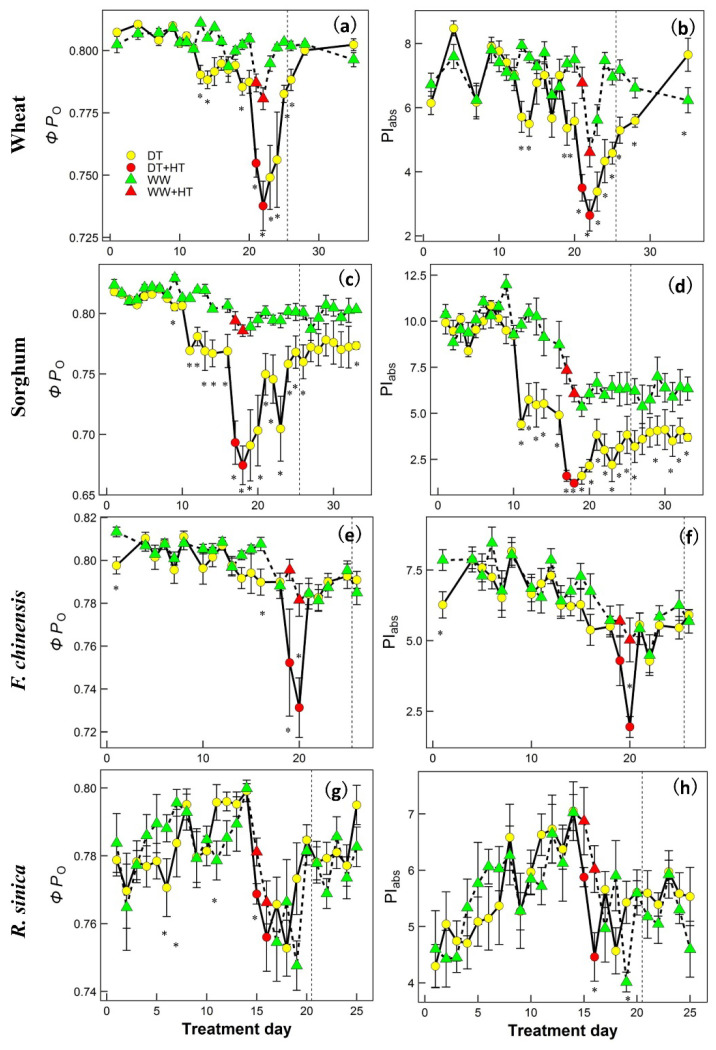
Time series responses of two representative chlorophyll fluorescence OJIP parameters: ABS/RC (absorption flux (Panels **a**,**c**,**e**,**g**)) and DI_O_/RC (dissipation flux (Panels **b**,**d**,**f**,**h**)) which were upregulated by drought and/or heat stress in wheat (**a**,**b**), sorghum (**c**,**d**), *F. chinensis* (**e**,**f**) and *R. sinica* (**g**,**h**). Plants were maintained under either drought (DT) or well-watered (WW) conditions and subjected to 2-day heat stress (HT) during the drought period. The dashed vertical line represents re-watering of the DT plants. Data show the mean ± S.E. of four replicates (n = 4). At each time point, ‘*’ indicates significant differences between the data for plants maintained under drought and well-watered conditions from one-way analysis of variance (*p* < 0.05).

**Figure 5:**
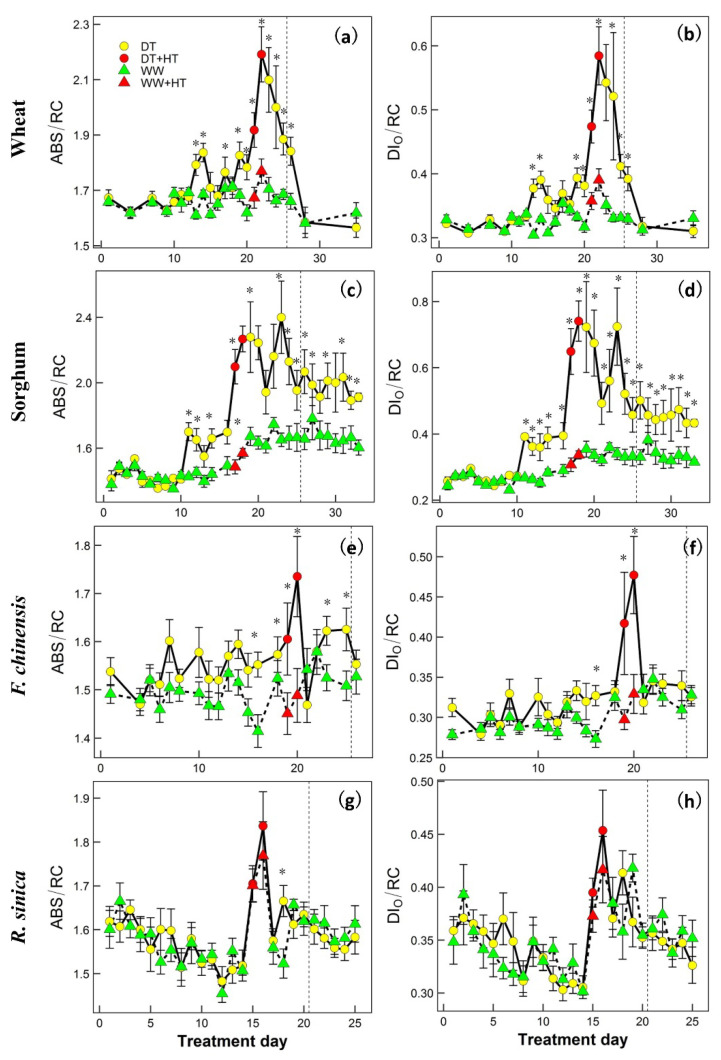
Time series responses of two representative chlorophyll fluorescence OJIP parameters: *ΦP*_O_ (quantum yield of primary photochemistry (Panels (**a**,**c**,**e**,**g**)) and PI_abs_ (performance index on an absorption basis (Panel (**b**,**d**,**f**,**h**)) which were downregulated by drought and/or heat stress in wheat (**a**,**b**), sorghum (**c**,**d**), *F. chinensis* (**e**,**f**) and *R. sinica* (**g**,**h**). Plants were maintained under either drought (DT) or well-watered (WW) conditions and subjected to 2-day heat stress (HT) during the drought period. The dashed vertical line represents re-watering of the DT plants. Data show the mean ± S.E. of four replicates (n = 4). At each time point, ‘*’ indicates significant differences between the data for plants maintained under drought and well-watered conditions from one-way analysis of variances (*p* < 0.05).

**Figure 6:**
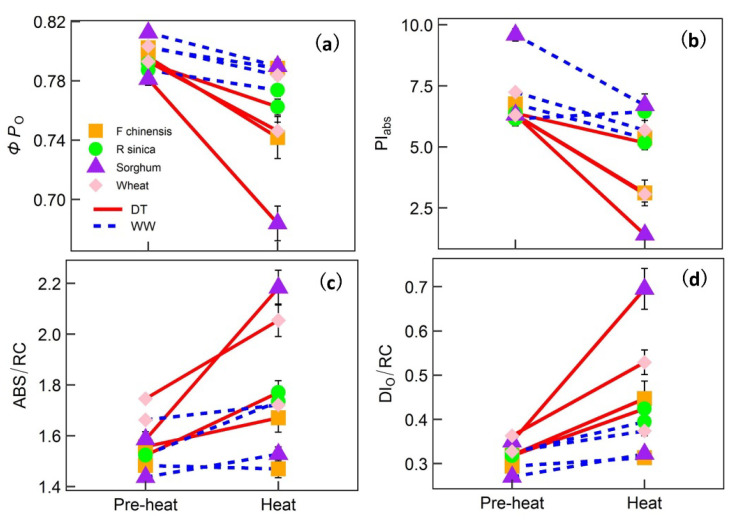
Responses of two downregulated parameters, *ΦP*_O_ (quantum yield of primary photochemistry (Panel (**a**)) and PI_abs_ (performance index on an absorption basis (Panel (**b**)), and two upregulated representative OJIP parameters, ABS/RC (absorption flux (Panel (**c**)) and DI_O_/RC (dissipation flux (Panel (**d**)) to either heat, drought or their combination in four plant species. The red line connects drought-treated (DT) plants of each species under the pre-heat and heat treatments; the blue dashed line connects well-watered (WW) plants of each species. The corresponding linear mixed analysis of variance results are shown in Table 2.

Figure 4 and Figure 5 show the time series for two representative upregulated parameters (*ΦP*_o_ and PI_abs_) and two downregulated OJIP parameters (ABS/RC and DI_o_/RC) for each species. To more effectively see how heat, drought and their combination affected the parameters, the response patterns for both WW and DT plants of all species in the pre-heat (which included data from the DT treatment phase) and heat phases are shown in Figure 6, and the corresponding linear mixed-effects analysis of variance results are shown in Table 2 and Table S1. In all species, heat alone had significant effects on *M*_o_, *ΔV*_IP_ (relative amplitude of the I–P phase), ABS/RC, DI_o_/RC, ET_o_/RC, RE_o_/RC, *ΦP*_o_, *ΦD*_o_; *ΦE*_o_, *ΦR*_o_, *φ*_o_, PI_abs_ and PI_tot_. The interaction between heat and species was significant for all OJIP parameters shown, indicating the effects on these parameters were species-dependent. Drought alone in all species significantly affected *V*_OJ_, ABS/RC, DI_o_/RC, ET_o_/RC, RE_o_/RC, *ΦP*_o_ and *ΦD*_o_. The interaction between drought and species for these affected parameters was also significant, suggesting that the magnitude of effects was species-dependent. The combination of heat with pre-existing drought exacerbated the effects (a significant interaction between heat and drought) on *M*_o_, *ΔV*_IP_, ABS/RC, DI_o_/RC, ET_o_/RC, RE_o_/RC, *ΦP*_o_, *ΦD*_o_, *ΦE*_o_, *ΦR*_o_, δ*R*_o_, *φ*_o_, PI_abs_ and PI_tot_ over heat or drought alone. In summary, heat and drought alone significantly affected the OJIP parameters and the effects were species-dependent; heat in combination with drought exacerbated the effects on quantum yield/efficiency, heat dissipation or flux and performance indices in all species.

After the relief of heat stress while the drought continued, DT plants showed the recovery of *M*_o_, *ΔV*_IP_, DI_o_/RC, ET_o_/RC, RE_o_/RC,*ΦP*_o_, *ΦD*_o_; *ΦE*_o_, *ΦR*_o_, *φ*_o_, PI_abs_ and PI_tot_ to a significant level (Table 3 and Table S2). Interestingly, no significant effects of heat relief on the ABS/RC of DT plants were found, and the interaction with species was not significant, indicating the lack of recovery from heat in effective antenna size in the reaction center while drought persisted. The effects of re-watering on DT plants were not significant but showed a significant interaction with species (Table S2), suggesting that not all species showed recovery after re-watering.

**Figure 7:**
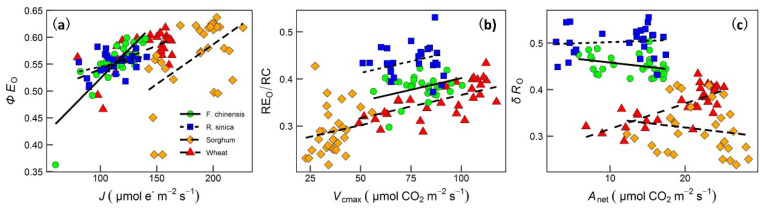
Linear regressions between photosynthetic traits reported in Zhu et al. [10,11] and chlorophyll fluorescence OJIP parameters. Panel (**a**) is for the modeled electron transport rate (*J*) vs. the quantum yield of electron transport (*ΦE*_o_) (*r*^2^ = 0.411, *p* < 0.0001; intercepts and slopes differed among species), Panel (**b**) is for the modeled maximum velocity of Rubisco carboxylation (*V*_cmax_) vs. electron flux leading to the reduction in the PSI end acceptor (RE_o_/RC) (*r*^2^ = 0.716, *p* < 0.0001; species shared similar slopes and different intercepts), Panel (**c**) is for net photosynthesis (*A*_net_) vs. the probability that an electron from the intersystem electron carriers is transported to the PSI end acceptor (*δR*_o_) (*r*^2^ = 0.823, *p* < 0.0001; intercepts and slopes differed among species). Linear regression results are shown in Table S3. *J* and *V*_cmax_ were modeled from net assimilation rate—intercellular CO_2_ concentration (*A*_net_—*C*_i_) curves, and *A*_net_ was measured under a light intensity of 1500 PPFD at 25 °C.

The linear relationships between the OJIP parameters and the measurements of *J* (modeled electron transport rate from the net assimilation rate—intercellular CO_2_ concentration (*A*_net_–*C*_i_) curves), *V*_cmax_ (modeled maximum velocity of Rubisco carboxylation from *A*_net_–*C*_i_ curves) and *A*_net_ under a light intensity of 1500 PPFD at 25 °C, which have been reported in our previous two companion studies [10,11], were also investigated (Figure 7, Table S3). *J* showed the strongest linear relationships (positive, *r*^2^ = 0.411, *p* < 0.0001) with the quantum yield of electron transport (*ΦE*_o_); the linear regressions differed in both their intercepts and slopes among the four species. *V*_cmax_ showed the strongest linear relationships (positive, *r*^2^ = 0.716, *p* < 0.0001) with electron flux leading to the reduction in the PSI end acceptor (RE_o_/RC); species shared similar slopes but differed in their intercepts. *A*_net_ showed the strongest linear relationships (positive for *R. sinica* and wheat; negative for *F. chinensis* and sorghum; *r*^2^ = 0.823, *p* < 0.0001) with the probability that an electron from the intersystem electron carriers would be transported to the PSI end acceptor (*δR*_o_). These significant linear relationships provide strong evidence for the utility of OJIP kinetics in predicting photosynthetic performance from electron transport between PSI and PSII to carboxylation of Rubisco and net CO_2_ assimilation.

## 3. Discussion

This study followed the day-to-day variation in OJIP chlorophyll fluorescence kinetics in four contrasting plant species, in response to a 2-day heat stress that was imposed during the middle of a 2-week drought. It was confirmed in the four contrasting plant species that a simulated heat wave (i.e., 2-day heat stress) further affected the OJIP kinetics, Figure 3, Figure 6 and Figure S1; Table 2, Table 3, Table S1 and Table S2), indicating enhanced impairment of multiple components of PSII function. This finding suggests the higher risk of future weather disasters impacting on plants’ photochemical performance, with climate change projected to give a more intense and longer duration of both heat and drought events [4,5].

Our study shows that heat, in combination with pre-existing drought, further affected many aspects of PSII, including specific fluxes (energy flux, transport flux, dissipation flux and electron flux) of the active PSII reaction center, quantum efficiency and efficiency of energy conservation. Jiang and Huang [9] reported that the combination of heat and drought were more detrimental to net photosynthesis (*A*_net_) and photochemical efficiency in Kentucky bluegrass than either stress alone. Similar results were also found in tomato plants [8]. Heat combined with pre-existing drought further increased the probability that the energy of an absorbed photon would be dissipated as heat, and the dissipation flux increased as well (an increase in *ΦD*_o_ and DI_o_/RC). The increase in heat dissipation, on one hand, suggests there was damage to the reaction centers (an increase in ABS/RC and a decrease in quantum yield; Figure 4 and Figure 6), thus yielding less energy for downstream photochemistry (a decrease in *ΦR*_o_ and *δR*_o_, Figure S1). On the other hand, an increase in heat dissipation has been recognized as an effective way for a plant to protect its thylakoid membranes from oxidative damage [40,41]. 

Our previous studies reported that in the four species, a 2-day heat stress, when applied on top of the pre-existing drought, did not reduce stomatal conductance or the photosynthetic rate, but a lower intrinsic water use efficiency was observed [10,11]. It was proposed that when heat stress occurred during the drought, transpirational cooling of the leaves was the priority over water conservation [42,43,44]. The exacerbated effects on OJIP observed in this study under heat stress reflect the potential damage to the PSII apparatus, and thus transpirational cooling of the CO_2_ and water exchange regulation, mediated through stomatal regulation, was likely to avoid more extensive damage. 

The decrease in quantum efficiency (indicated by *ΦP*_o_, *ΦE*_o_, *ΦR*_o_, *φ*_o_, PI_abs_ and PI_tot_) and the increase in heat dissipation (indicated by *ΦD*_o_ and DI_o_/RC) induced by high temperature and/or drought was consistently found in the current study, while some discrete patterns between heat and drought, or among species, have emerged for some components related to electron transport or flux. In a few previous studies, the K peak (indicated by *V*_OJ_ in our study) has been found to be induced by heat or drought [16,24,25,33,34]. The presence of the K peak indicates the inhibition of electrons donated by the oxygen-evolving complex. Here, it was induced in all species by drought, and by heat in three species but not *F. chinensis* (Figure S1, Table S2). Interestingly, the effect of heat stress on the tropical tree species *R. sinica* was in the opposite direction to that of the other three species in respect to some OJIP parameters (Figure S1, ET_o_/RC, RE_o_/RC, *δR*_o_). In the other three species, both heat and/or drought reduced these parameters; however, in *R. sinica*, drought reduced while heat increased these OJIP parameters for both WW and DT plants. This contrary response pattern might be because this tropical tree species has adapted to hot/warm weather. The high temperature in our protocol (40 °C) may be considered as moderate for this species, potentially benefiting PSII components that are related to electron transport (ET_o_/RC) or electron flux (RE_o_/RC) or both (*δR*_o_). 

Species also showed differences in the recovery phase. The heat-induced effects in the DT wheat and DT sorghum showed slower recovery in ABS/RC, *ΦP*_o_ and PI_abs_ than those of *F. chinensis* and *R. sinica* (Figure 4 and Figure 5, Table 2 and Table 3). The two tree species actually had lower stomatal conductance, indicating a higher level of drought response as compared with that experienced by the two crop species during the drought period. However, the OJIP parameters of the two tree species decreased at a slower rate. This contrary pattern suggests that the stomatal regulation of the two tree species was more sensitive to drought, leading to a faster or a greater rate of stomatal closure, but maintained better PSII function, which resulted in faster recovery ability when heat was released [45]. 

The recovery of most OJIP parameters after heat or drought relief seemed to have progressed well, with ABS/RC as an exception (Figure 4 and Figure 5; Table 3 and Table S2). ABS/RC failed to recover to the pre-heat level after the relief of heat before re-watering in all species; sorghum did not recover even after re-watering for 8 days (Figure 4 and Figure 5; Table 3 and Table S2). The increase in ABS/RC has commonly been found after plants have been subjected to either increased temperature [24,33,37] or drought [26,28]. The increase in ABS/RC indicates a decrease in the size of the chlorophyll antenna serving each reaction center [31,33]. It is likely that once the chlorophyll antenna has been damaged, full recovery cannot be achieved. In addition to the interesting biological indications inferred for the recovery phase, monitoring the OJIP kinetics during the recovery from either drought or heat also proved effective for predicting plant productivity.

The strong linear relationships between photosynthesis traits (*J*—modeled electron transport rate from net assimilation rate—intercellular CO_2_ concentration (*A*_net_—*C*_i_) curves; *V*_cmax_—modeled maximum velocity of Rubisco carboxylation *A*_net_—*C*_i_ curves; *A*_net_ under a light intensity of 1500 PPFD at 25 °C) and some OJIP parameters (Figure 7) suggested the possibility of predicting the growth potential using OJIP kinetics. Galic et al. [27] found that plant biomass in salt-stressed young maize plants can be modeled by photosynthetic performance. It is likely therefore that plant productivity under various abiotic conditions could be predicted using OJIP kinetics. It is worth pointing out that combining measurements of OJIP kinetics with artificial intelligence [46] and remote sensing [47,48] may benefit agricultural and forest management. Especially during extreme weather seasons, the chlorophyll fluorescence OJIP kinetics could be a very useful and non-invasive tool for monitoring plant performance and would help with the prediction of primary productivity in crop growth or vegetation. 

## 4. Materials and Methods

### 4.1. Plant Materials and Growth Conditions

**Figure 8:**
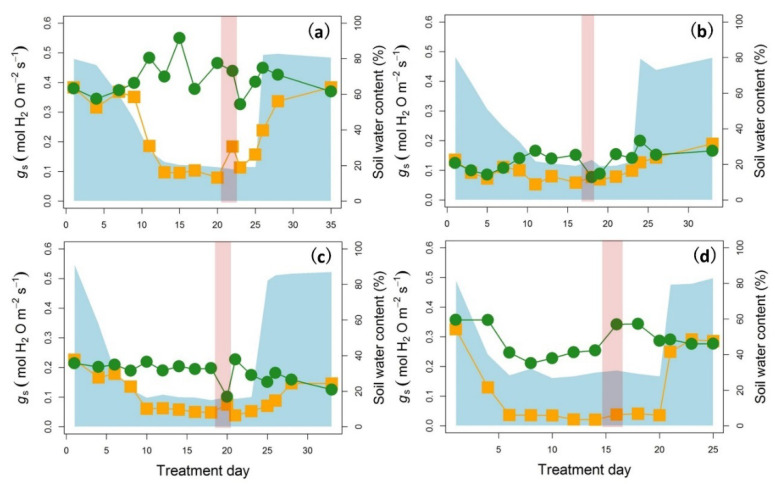
Time courses of stomatal conductance (*g*_s_) and soil water content for wheat (**a**), sorghum (**b**), *F. chinensis* (**c**) and *R. sinica* (**d**). Green circles represent plants always under well-watered conditions. Data show the mean of four replicates (n = 4). Orange squares represent plants subjected to drought treatment. The blue shaded area indicates the soil water content and the pink shaded area indicates the 2-day heat stress, in which both day and night temperatures were increased by 15 °C.

This study shared the same plant materials and treatments as described by Zhu et al. [10,11]. Experiments were conducted on wheat from September to December 2018, on sorghum from June to October 2018, on *F. chinensis* from May to September 2018 and on *R. sinica* from July 2018 to January 2019. The general experimental routine for the four species is as follows: drought was firstly initiated by withholding water and, in the meantime, stomatal conductance (*g*_s_) was monitored, i.e., using *g*_s_ as a drought indicator [49] (Figure 8a–d). When *g*_s_ had achieved the expected value range, we made an effort to maintain the soil water content to maintain a similar drought level. When the drought level had lasted for about 1 week, a 2-day heat stress was imposed by elevating the temperature by 15 °C. After relief of the heat stress, the drought level continued for about 5 days before re-watering (Figure 8e–h). For detailed growth conditions for the four species, refer to Zhu et al. [10,11].

The definition of drought was based on monitoring the stomatal conductance of an intact leaf using a LI-6400 (Li-Cor 6400; Li-Cor Inc., Lincoln, NE, USA) every 2 days (Figure 1e–h). Soil water content (SWC) for wheat and sorghum was calculated as the ratio of actual water content and the potential available soil water content, which was defined as the weight loss of fully water-saturated soil in pots after drying for 2 days at 105 °C [50]. The SWC of *F. chinensis* and *R. sinica* was measured by a portable soil moisture sensor (Kaiouya, Wuxi, China), which was calibrated against a known SWC.

Wheat plants were grown in growth chambers at 25/20 °C (day/night) with a 14 h day length. Relative humidity was 50% and light intensity was ~450 µmol m^−2^ s^−1^ at the top canopy leaf level. Twenty-eight plants were grown in two identical growth chambers with the same settings: 14 plants for the drought treatment (DT plants) and the other 14 always under well-watered conditions (WW plants). In each chamber, 7 DT plants and 7 WW plants were randomly located. The experiment on sorghum followed the same design as that for wheat. The growth chamber settings for sorghum were 28/23 °C (day/night) with a 12 h day and a 12 h night, 50% relative humidity and 450 µmol m^−2^ s^−1^ light intensity at the top canopy level. 

For *F. chinensis* and *R. sinica*, both tree species were initially grown outdoors for 2 months. When they reached ~50 cm in height, they were moved into two growth chambers. The four species shared the same two growth chambers. All plants received adequate irrigation for 2 weeks in order to acclimate to the new growth conditions before the measurement period commenced. For each species, eight tree seedlings were randomly located in two growth chambers. The temperature setting was 25/20 °C (day/night), a 14 h day length, 50% relative humidity and 450 µmol quantum m^−2^ s^−1^ light intensity at the top canopy. Four DT plants and four WW plants were selected. No water was added to the DT pots until *g*_s_ reduced to ~0.05 mol H_2_O m^−2^ s^−1^*,* and then the SWC was maintained. For all four species, the SWC was maintained by weighing the pots by only adding the amount of water that compensated the amount that had been evaporated the previous day.

### 4.2. Chlorophyll Fluorescence Measurements

The OJIP kinetics (Table 1 lists the parameters and their calculations) were measured on the first youngest fully expanded leaves at about 1 hour after chamber light was switched off. The plants were taken out from the growth chamber and the chlorophyll fluorescence rise was measured at room temperature (~25 °C) on attached leaves. The measurement location was at about one-third from the tip of the leaf, avoiding the middle vein for wheat and sorghum; for the two tree species, it was at the middle part, avoiding the middle vein. The chlorophyll fluorescence OJIP kinetics were assessed using a portable fluorimeter (PSI FluorPen FP100, Brno, Czech Republic). For each measurement, a detachable leaf clip was mounted on one leaf and the Fluorpen was mounted vertically on the leaf clip surface. The fluorescence signal was recorded with a time resolution of 10 μs. At the start of the measurements, weak light (2–3 µmol quantum m^−2^ s^−1^) was shone on the upper epiderma for steady-state basal fluorescence. To induce the samples, a saturating light pulse of 3500 µmol quantum m^−2^ s^−1^ was provided with a peak wavelength of 627 nm for 1 s. 

### 4.3. Statistics

Specific contrasts based on experimental sequential phases were set up in order to test different treatment effects. The categorization of experimental sequential phases was: (1) drought initiation (Day 1—the day before the start of targeted drought treatment), (2) drought (the start of targeted drought stress to the start of the heat treatment: Days 11–20 for wheat, Days 10–16 for sorghum, Days 10–18 for *F. chinensis* and Days 8–14 for *R. sinica*), (3) heat or heat combined with drought (2-day heat stress: Days 21–22 for wheat, Days 17–18 for sorghum, Days 19–20 for *F. chinensis* and Days 15–16 for *R. sinica*), (4) heat removal (the day after the relief of heat stress and before re-watering: Days 23–25 for wheat, Days 19–23 for sorghum, Days 21–25 for *F. chinensis* and Days 17–20 for *R. sinica*) and, finally, (5) re-watering. First, to test the effects of heat combined with drought, a three-way analysis of variance (ANOVA) based on a linear mixed-effects model was constructed, with heat, drought, species and their interactions as fixed effects, and individual plants as random effects. In the linear mixed-effects model, heat included the 2-day data of all species; drought included the start of targeted drought stress to the start of the heat treatment. To test the effects of heat release on either WW or DT plants, heat relief was tested against heat, and the interaction with species (two-way ANOVA) was included as a fixed effect and individual plants as random effects. To test the effects of re-watering on DT plants, re-watering was tested against heat relief in DT plants, and the interaction with species was included as a fixed effect and individual plants as random effects. One-way ANOVA was applied to compare the differences at each time point, and the differences between DT and WW plants. Principal component analysis (PCA) was performed to investigate the distribution patterns of the OJIP parameters and treatments. Statistical analyses were conducted using R [51].

## 5. Conclusions

In conclusion, by measuring the biophysical response of photosystem II (PSII) to a 2-day heat stress during a 2-week drought, using the OJIP kinetics, this study confirmed, in four contrasting plant species, that the combination of heat stress with pre-existing drought resulted in enhanced impairment of multiple components of PSII function. This included decreased quantum efficiency and increased heat dissipation. Divergent patterns in some components related to electron transport or flux between heat and drought, and among species were found. The significant linear relationships between the OJIP parameters and *J*, *V*_cmax_ and *A*_net_ suggest that utility of OJIP kinetics is a prime candidate for predicting these three aspects of photosynthetic performance, from electron transport between PSI and PSII to carboxylation of Rubisco and net CO_2_ assimilation. These findings point to future research and the application of chlorophyll fluorescence OJIP kinetics in agriculture and forestry for facing climate change.

## Figures and Tables

**Table 1:** Definitions and explanations of selected chlorophyll fluorescence OJIP parameters in this study. The parameters are derived from the literatures [19,26,39].

Fluorescence Parameters		Calculation
***F*_o_**	Fluorescence signal at 50 μs	-
*F*_300_	Fluorescence signal at 300 μs	-
*F*_J_	Fluorescence signal at 2 ms	-
*F*_I_	Fluorescence signal at 30 ms	-
*F*_M_	Maximal fluorescence signal	-
*M*_o_	Initial slope of the induction curve	4 (*F*_300_ − *F*_o_)/(*F*_M_ − *F*_o_)
*V*_J_	Relative variable fluorescence at 2 ms	(*F*_J_ − *F*_o_)/(*F*_M_ − *F*_o_)
*V*_I_	Relative variable fluorescence at 30 ms	(*F*_I_ − *F*_o_)/(*F*_M_ − *F*_o_)
*V*_OJ_	Relative variable fluorescence of the O–J phase at 300 μs	(*F*_300_ − *F*_o_)/(*F*_J_ − *F*_o_)
Δ*V*_IP_	Relative amplitude of the I–P (*F*_M_) phase	(*F*_M_ − *F*_I_)/(*F*_M_ − *F*_o_)
*Specific fluxes per active PSII reaction center*		
ABS/RC	Absorption flux, effective antenna size of an active reaction center	*M*_o_ (1/*V*_J_) (1/*ΦP*_o_)
TR_o_/RC	Trapped energy flux leading to a reduction in *Q*_A_	*M*_o_ (1/*V*_J_)
ET_o_/RC	Electron transport flux further than *Q*_A_	*M*_o_ (1/*V*_J_) (1 − *V*_J_)
DI_o_/RC	Dissipation flux	ABS/RC − TR_o_/RC
RE_o_/RC	Electron flux leading to a reduction in the PSI end acceptor	*M*_o_ (1/*V*_J_) (1 − *V*_I_)
*Quantum efficiency/flux ratios*		
*ΦP*_o_	Quantum yield of primary photochemistry; probability that an absorbed photon leads to a reduction in *Q*_A_	(*F*_M_ − *F*_o_)/*F*_M_
*ΦE*_o_	Quantum yield of electron transport; probability that an absorbed photon leads to electron transport further than *Q*_A_	ET_o_/ABS
*ΦD*_o_	Probability that the energy of an absorbed photon is dissipated as heat	1 − *ΦP*o
*ΦR*_o_	Probability that an absorbed photon leads to a reduction in the PSI end acceptor	RE_o_/ABS
*δR*_o_	Probability that an electron from the intersystem electron carriers is transported to the PSI end acceptor	RE_o_/ET_o_
*φ*_o_	Probability that an absorbed photon leads to reduction further than *Q*_A_.	1 − *V*_J_
*Area above the induction curve*		
Area	Integrated area between the induction curve and *F*	*F*_M_
*S_M_*	Normalized area	Area/(*F*_M_ − *F*_o_)
*Performance indices*		
PI_abs_	Performance index on an absorption basis: the efficiency of energy conservation from absorbed photons to a reduction in intersystem electron carriers	(RC/ABS) [(*ΦP*o/(1 − *ΦP*o)] [(1 − *V*_J_)/(1 − (1 − *V*_J_))]
PI_tot_	Efficiency of energy conservation from the absorbed photons to a reduction in PSI end acceptors	PI_abs_ *δR*o/(1 − *δR*o)

**Table 2:** Results of linear mixed-effects analysis of variance on the effects of heat stress combined with pre-existing drought.

Treatments	*ΦP*_O_		PI_abs_		ABS/RC		DI_O_/RC	
	t Value	*p* Value	t Value	*p* Value	t Value	*p* Value	t Value	*p* Value
Heat	−12.449	**<0.0001**	−13.632	**<0.0001**	3.332	**0.0009**	7.824	**<0.0001**
Drought	2.136	**0.033**	1.653	0.0988	−4.279	**<0.0001**	−3.625	**0.0003**
Species	−3.386	**0.0007**	6.726	**<0.0001**	9.705	**<0.0001**	5.727	**<0.0001**
Heat × Drought	6.734	**<0.0001**	6.652	**<0.0001**	−2.859	**0.004**	−4.478	**<0.0001**
Heat × Species	−4.765	**<0.0001**	−2.981	**0.003**	7.109	**<0.0001**	5.999	**<0.0001**
Drought × Species	4.414	**<0.0001**	4.427	**<0.0001**	−2.713	**0.0068**	−4.324	**<0.0001**
Heat × Drought × Species	2.779	**0.006**	−2.574	**0.010**	−3.638	**0.0003**	−3.024	**0.002**

Note: Values in bold indicate significant response at significance level of *p*-value < 0.05.

**Table 3:** Results of linear mixed-effects analysis of variance on the effects of heat stress combined with pre-existing drought.

Treatments	*ΦP*_O_		PI_abs_		ABS/RC		DI_O_/RC	
	t Value	*p* Value	t Value	*p* Value	t Value	*p* Value	t Value	*p* Value
Heat Relief (DT)	4.971	**<0.0001**	6.531	**<0.0001**	−1.692	0.092	−3.587	**0.0004**
Species	−2.384	**0.018**	−1.864	0.0640	5.651	**<0.0001**	3.949	**0.0001**
Heat Relief (DT) × Species	−1.873	0.0630	−3.955	**0.0001**	0.097	0.922	1.034	0.302
Heat Relief (WW)	−0.283	0.777	0.712	0.477	2.583	**0.010**	1.619	0.107
Species	−2.468	**0.014**	2.689	**0.008**	6.950	**<0.0001**	5.258	**<0.0001**
Heat Relief (WW) × Species	2.544	**0.012**	−2.223	**0.027**	−4.517	**<0.0001**	−2.944	**0.004**
Re-watering (DT)	−0.772	0.441	−1.181	0.239	0.601	0.548	0.861	0.390
Species	−2.303	**0.022**	−3.518	**0.001**	6.166	**<0.0001**	4.718	**<0.0001**
Re-watering (DT) × Species	−2.499	**0.013**	−2.891	**0.004**	4.401	**<0.0001**	3.696	**0.0003**

Note: Values in bold indicate significant response at significance level of *p*-value < 0.05.

## Data Availability

The data presented in this study are available in the Supplementary Material as Excel files.

## Supplementary Materials

The following are available online at https://www.mdpi.com/article/10.3390/ijms221910682/s1.
