# Supplementary material for: Combining Heat Stress with Pre-Existing Drought Exacerbated the Effects on Chlorophyll Fluorescence Rise Kinetics in Four Contrasting Plant Species
Source: Int J Mol Sci. 2021 Oct 1;22(19):10682. doi: 10.3390/ijms221910682 (PMC8508795; doi:10.3390/ijms221910682)
Supplement: Supplementary file 1 [file ijms-22-10682-s001.zip › ijms-1371112-supplementary/Supplementary material.pdf]

Supporting information for

Combining heat stress with pre-existing drought exacerbated effects on chlorophyll fluorescence rise kinetics in four contrasting plant species

Authors: Lingling Zhu, Wei Wen, Michael R. Thorpe, Charles H. Hocart, Xin Song

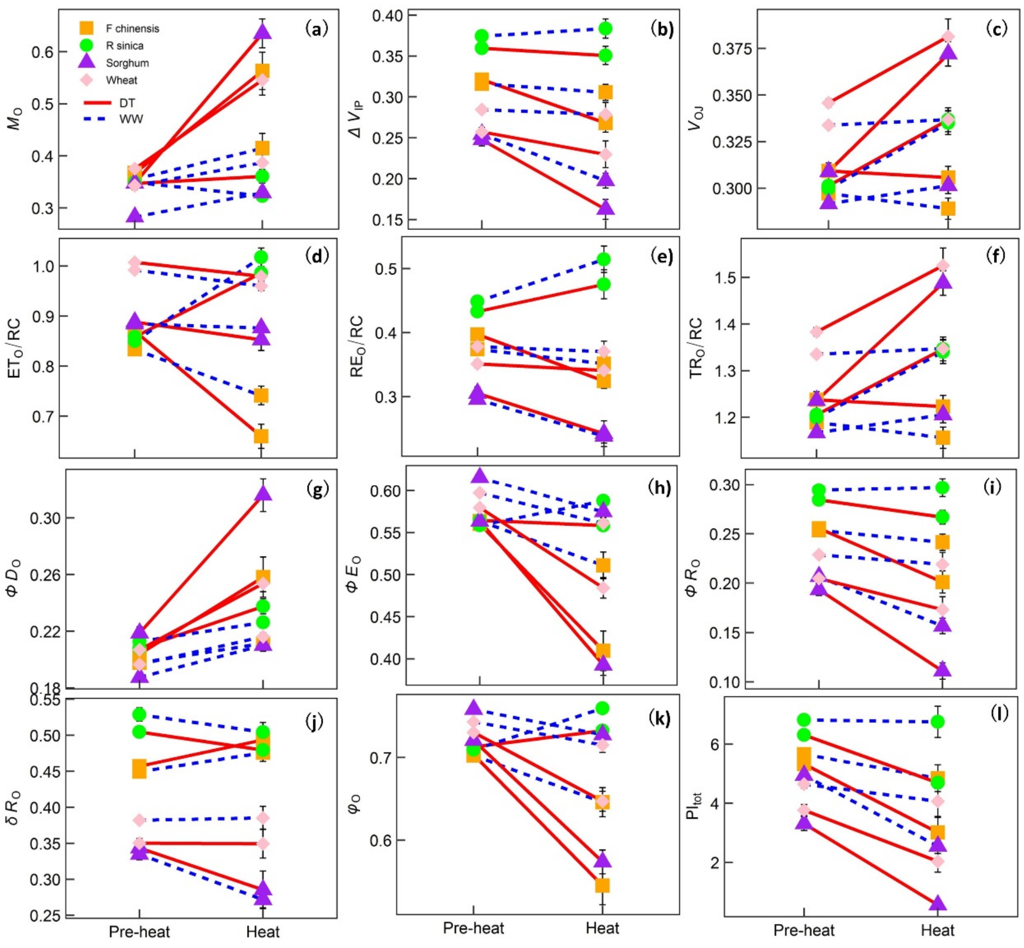

Figure S1. Responses of chlorophyll fluorescence OJIP parameters to either heat, drought or their combination in four plant species. Red line connects each drought-treated (DT) species under pre-heat and heat treatments; blue dashed line connects each well-watered (WW) species. The corresponding linear mixed-effects model analysis of variances results are shown in Table S3.

Table S1. Linear mixed-effects model analysis of variance of the effects of heat and drought and their interaction on OJIP parameters.

		Heat	Drought	Species	Heat x Drought	Heat x Species	Drought x Species	Heat x Drought x Species
*M*o	t value	10.482	-1.364	-1.783	-5.041	-5.95	-3.686	-1.838
	P value	**< 0.0001**	0.173	0.075	**< 0.0001**	**< 0.0001**	**0.0002**	0.066
V_{OJ}	t value	-1.005	-4.422	11.867	-0.780	6.896	2.377	-3.385
	P value	0.315	**< 0.0001**	**< 0.0001**	0.435	**< 0.0001**	**0.018**	**0.0007**
ΔV_{IP}	t value	-4.951	-0.655	-9.138	2.814	2.650	3.794	-1.168
	P value	**< 0.0001**	0.513	**< 0.0001**	**0.005**	**0.008**	**0.0001**	0.243
TRo/RC	t value	-1.02	-4.444	11.838	-0.767	6.896	2.390	-3.388
	P value	0.308	**< 0.0001**	**< 0.0001**	0.443	**< 0.0001**	**0.017**	**0.0007**
ETo/RC	t value	-17.693	-3.916	14.612	7.185	16.312	1.820	-3.354
	P value	**< 0.0001**	**< 0.0001**	**< 0.0001**	**< 0.0001**	**< 0.0001**	0.069	**0.0008**
REo/RC	t value	-6.031	-2.572	-7.732	2.861	5.238	4.550	1.739
	P value	**<0.0001**	**0.01**	**< 0.0001**	**0.004**	**< 0.0001**	**< 0.0001**	0.082
*ΦE*o	t value	-17.579	0.639	2.883	8.517	10.650	3.448	-3.926
	P value	**< 0.0001**	0.523	**0.004**	**< 0.0001**	**< 0.0001**	**0.0006**	**< 0.0001**
*ΦD*o	t value	11.109	-2.503	3.974	-5.440	4.152	-5.300	-2.046
	P value	**< 0.0001**	**0.012**	**< 0.0001**	**< 0.0001**	**< 0.0001**	**< 0.0001**	**0.041**
Φ*R*o	t value	-6.512	-0.308	-8.742	3.643	-3.46	3.682	-1.492
	P value	**< 0.0001**	0.758	**< 0.0001**	**0.0003**	**0.00005**	**0.0002**	0.136
δ*R*o	t value	2.325	-0.761	-12.44	-0.519	-4.032	3.797	0.699
	P value	**0.02**	0.446	**< 0.0001**	0.603	**< 0.0001**	**0.0002**	0.484

φo	t value	-17.385	0.014	4.004	8.131	11.961	2.647	-3.911
	P value	**< 0.0001**	0.988	**< 0.0001**	**< 0.0001**	**< 0.0001**	**0.008**	**0.0001**
PI_{tot}	t value	-12.384	1.491	-2.048	6.021	6.04	4.016	-2.213
	P value	**< 0.0001**	0.136	**0.041**	**< 0.0001**	**< 0.0001**	**< 0.0001**	**0.027**

Table S2. Linear mixed-effects model analysis of variance of the effects of heat release and re-watering on OJIP parameters.

		Heat release (DT)	Species	Heat release (DT) x Species	Heat release (WW)	Species	Heat release (WW) x Species	Re-watering (DT)	Species	Re-watering (DT) x Species
*M*o	t value	-5.616	-5.771	3.417	-0.625	-3.744	1.732	1.104	4.431	3.213
	P value	**< 0.0001**	**< 0.0001**	**0.0007**	0.532	**0.0002**	0.085	0.27	**< 0.0001**	**0.005**
V_{OJ}	t value	0.542	4.982	-2.596	2.956	8.209	-5.895	0.436	7.129	4.704
	P value	0.588	**< 0.0001**	**0.01**	**0.004**	**< 0.0001**	**< 0.0001**	0.663	**< 0.0001**	**< 0.0001**
ΔV_{IP}	t value	2.993	-5.473	-2.233	1.162	-4.973	-1.490	-0.805	-9.419	2.967
	P value	**0.003**	**< 0.0001**	**0.027**	0.247	**< 0.0001**	0.138	0.421	**< 0.0001**	**0.003**
TRo/RC	t value	0.553	3.441	4.978	2.951	8.207	-5.892	0.441	7.122	4.701
	P value	0.58	**0.0007**	**< 0.0001**	**0.004**	**< 0.0001**	**< 0.0001**	0.66	**< 0.0001**	**< 0.0001**
ETo/RC	t value	9.911	12.897	-8.879	4.991	13.597	-9.091	-0.807	6.374	3.915
	P value	**< 0.0001**	**< 0.0001**	**< 0.0001**	**< 0.0001**	**< 0.0001**	**< 0.0001**	0.42	**< 0.0001**	**< 0.0001**
REo/RC	t value	3.571	5.839	-3.395	2.647	8.363	-4.323	-0.615	-6.48	3.482
	P value	**< 0.0001**	**< 0.0001**	**0.0008**	**0.009**	**< 0.0001**	**< 0.0001**	0.538	**< 0.0001**	**0.0006**
*ΦE*o	t value	8.332	7.54	4.716	1.778	4.912	-3.387	-1.272	-2.118	-1.858
	P value	**< 0.0001**	**< 0.0001**	**< 0.0001**	0.77	**< 0.0001**	**0.0008**	0.204	**0.035**	0.064
*ΦD*o	t value	-4.931	3.047	2.532	0.409	2.733	-2.852	1.017	2.735	2.592
	P value	**< 0.0001**	**0.003**	**0.012**	0.683	**0.001**	**0.004**	0.31	**0.007**	**0.01**
Φ*R*o	t value	3.789	-5.498	-2.49	1.109	-4.682	-1.674	-0.913	-9.288	-2.412
	P value	**0.0002**	**< 0.0001**	**0.014**	0.269	**< 0.0001**	0.095	0.362	**< 0.0001**	**0.017**
δ*R*o	t value	-1.466	-7.639	2.549	0.063	1.402	-8.646	-0.294	-10.095	3.447

	P value	0.144	**< 0.0001**	**0.012**	0.95	0.163	**< 0.0001**	0.769	**< 0.0001**	**0.0006**
φo	t value	9.060	9.485	6.335	2.188	6.498	-3.575	-1.357	-1.85	1.537
	P value	**< 0.0001**	**< 0.0001**	**< 0.0001**	**0.03**	**< 0.0001**	**0.0004**	0.176	0.066	0.126
PI_{tot}	t value	6.175	4.804	-3.78	0.722	2.445	-2.16	-1.155	-4.898	-3.112
	P value	**< 0.0001**	**< 0.0001**	**0.0002**	0.471	**0.015**	**0.032**	0.249	**< 0.0001**	**0.002**

Table S3. Linear regression results between ΦE_o and J, RE_o/RC and V_{cmax}, δR_o and A_{net}.

Linear model	a	b	r^2	P
$\Phi E_o = a + b * J$			0.4110	< 0.0001
Wheat	0.4533	0.0009		
Sorghum	0.2913	0.0015		
F. chinensis	0.3103	0.0021		
R. sinica	0.4983	0.0005		
$RE_o/RC = a + b * V_{cmax}$		0.001	0.7155	< 0.0001
Wheat	0.2674			
Sorghum	0.2525			
F. chinensis	0.3030			
R. sinica	0.3636			
$\delta R_o = a + b * A_{net}$			0.8232	< 0.0001
Wheat	2.61E-01	-2.17E-01		
Sorghum	3.56E-01	-1.89E-03		
F. chinensis	4.76E-01	-1.82E-03		
R. sinica	4.96E-01	5.52E-04		
